# Comparison of two elastic motion correction approaches for whole-body PET/CT: motion deblurring vs gate-to-gate motion correction

**DOI:** 10.1186/s40658-020-0285-4

**Published:** 2020-03-30

**Authors:** Stefanie Pösse, Florian Büther, Dirk Mannweiler, Inki Hong, Judson Jones, Michael Schäfers, Klaus Peter Schäfers

**Affiliations:** 1 European Institute for Molecular Imaging, University of Münster, Waldeyerstr. 15, Münster, 48149 Germany; 2 Department of Nuclear Medicine, University Hospital of Münster, Albert-Schweitzer-Campus 1, Münster, 48149 Germany; 3 Molecular Imaging, Siemens Medical Solutions Inc., Knoxville, Knoxville, USA

**Keywords:** Motion correction, Image reconstruction, PET/CT, Optical flow

## Abstract

**Background:**

Respiratory motion in PET/CT leads to well-known image degrading effects commonly compensated using elastic motion correction approaches. Gate-to-gate motion correction techniques are promising tools for improving clinical PET data but suffer from relatively long reconstruction times. In this study, the performance of a fast elastic motion compensation approach based on motion deblurring (DEB-MC) was evaluated on patient and phantom data and compared to an EM-based fully 3D gate-to-gate motion correction method (G2G-MC) which was considered the gold standard.

**Methods:**

Twenty-eight patients were included in this study with suspected or confirmed malignancies in the thorax or abdomen. All patients underwent whole-body [^18^F]FDG PET/CT examinations applying hardware-based respiratory gating. In addition, a dynamic anthropomorphic thorax phantom was studied with PET/CT simulating tumour motion under controlled but realistic conditions. PET signal recovery values were calculated from phantom scans by comparing lesion activities after motion correction to static ground truth data. Differences in standardized uptake values (SUV) and metabolic volume (MV) between both reconstruction methods as well as between motion-corrected (MC) and non motion-corrected (NOMC) results were statistically analyzed using a Wilcoxon signed-rank test.

**Results:**

Phantom data analysis showed high lesion recovery values of 91% (2 cm motion) and 98% (1 cm) for G2G-MC and 83% (2 cm) and 90% (1 cm) for DEB-MC. The statistical analysis of patient data found significant differences between NOMC and MC reconstructions for SUV _max_, SUV _mean_, MV, and contrast-to-noise ratio (CNR) for both reconstruction algorithms. Furthermore, both methods showed similar increases of 11–12% in SUV _max_ and SUV _mean_ after MC. The statistical analysis of the MC/NOMC ratio found no significant differences between the methods.

**Conclusion:**

Both motion correction techniques deliver comparable improvements of SUV _max_, SUV _mean_, and CNR after MC on clinical and phantom data. The fast elastic motion compensation technique DEB-MC may thereby be a valuable alternative to state-of-the art motion correction techniques.

## Background

PET/CT has evolved into a very sensitive and accurate technique for tumour diagnosis and staging. The reasons for this clinical success are manifold: (1) PET/CT is a hybrid technique combining functional and anatomical imaging within a single examination, (2) there exists a broad spectrum of tumour markers such as the glucose analogon ^18^F-FDG, and (3) PET/CT is a whole-body imaging method providing information not only in the region of interest, but also in the entire body. At the same time, PET has continuously been improved from a low-sensitive 2D imaging technology with poor spatial resolution into a high-resolution 3D technique with excellent signal-to-noise (SNR) characteristics using time-of-flight (TOF) capabilities [1–3]. This enabled a significant reduction in acquisition time to few minutes per bed position. During the acquisition period, however, patient motion may influence the acquired data leading to blurring and other types of motion-related artefacts.

Typical patient motion consists of physiological respiratory and cardiac motion, both being nearly periodical, bulk body motion, and non-periodical motion of the stomach and bowel system. For oncology examinations using PET imaging, respiratory motion has the highest impact on abdomino-thoracic tumours given the fact that motion could become quite large in individual subjects. Motion amplitudes of up to 6 cm in cranio-caudal direction have been measured within the lung, although the mean motion amplitude is typically in the order of 1 cm for normal patients [4]. Given the high spatial resolution of modern whole-body PET systems of 4–5 mm full width at half maximum (FWHM) [1, 5], respiratory motion is not only affecting tumour PET imaging, but has also a negative impact on quantitative imaging of organs like the liver, kidneys, or spleen [6]. To overcome this, different motion correction (MC) approaches have been proposed before which compared for example gated (amplitude-based or phase-based) to non-gated reconstructions or optimal respiratory-gating 4D PET/CT to 3D PET/CT [7–11].

The most straightforward correction method is to accept only data from the end-expiration phase of the breathing cycle and to reconstruct these data into a single 3D image. Although this technique, known as optimal gating (OG), provides almost motion-free images, a substantial fraction of the acquired data is discarded leading to higher noise levels and reduced image quality. If not compensated for by increasing the PET acquisition time, this leads to biassed SUVs.

More advanced MC techniques have been introduced taking into account all acquired data and performing a single image reconstruction by incorporating elastic transformations to deform the lines-of-response (LOR) according to a determined motion vector field (MVF) [12–15]. These methods are usually based on gated sinograms allowing to perform a gate-to-gate motion correction. Even more advanced methods of PET motion correction have been introduced to apply non-rigid corrections during image reconstruction on an event-to-event basis [16]. As the advanced MC techniques make use of all acquired data, the resulting image quality is comparable to static image reconstructions using the same raw data. In consequence, SUVs from advanced MC techniques are less biassed than OG derived SUVs due to reduced image noise. Therefore, advanced MC techniques are preferable to OG methods in clinical PET leading to reduced scanning times and enhanced SNR properties.

These techniques are slowly making their way into clinical practice but are still under development and are not yet completely validated and generally accepted methods. One reason is the fact that elastic motion correction is usually time-consuming and requires far more computational resources than ordinary image reconstructions.

In this study, a fast elastic motion compensation approach based on motion deblurring (DEB-MC) is evaluated for clinical usage. DEB-MC has already been evaluated before showing its potential of performing whole-body PET motion correction comparable to OG results [17]. However, as OG cannot be regarded as the gold standard for motion correction, we aim here for a systematic comparison of DEB-MC with an established gate-to-gate elastic motion compensation technique (G2G-MC) within a clinical patient cohort and advanced phantom scans. This MC method, already implemented in some clinical systems, can be considered as the current reference MC method as it provides superior image quality over simple gating methods like OG in terms of noise. Depending on the actual gating scheme, it is also expected to have sufficiently good motion resolution characteristics. Nevertheless, computation efforts for this method is high, while DEB-MC requires much less effort. If DEB-MC demonstrates similar performance as compared to G2G-MC, DEB-MC could therefore be a valuable option towards broader acceptance of elastic motion correction within a clinical PET/CT environment.

## Materials and methods

### Patient and phantom data

Twenty-eight patient datasets (57.6 ± 11.7 years and 74.1 ± 14.8 kg) with a total of 107 identified lesions were included in this retrospective comparative analysis of the MC approaches. Besides patient data, the dynamic anthropomorphic thorax phantom “Wilhelm” was used to evaluate the different MC strategies in a controlled situation and to compare results with ground truth (GT) data without any motion [18]. The phantom was prepared to simulate a human ^18^F-FDG study with a single lesion (0.25 ml volume) at the right diaphragm position. The lesion was able to move in cranio-caudal direction driven by a pneumo-hydraulic system which also compresses the lung inserts during expiration. The activity concentrations in the different compartments at the beginning of the GT phantom scan were 11.0 kBq/ml (heart), 3.7 kBq/ml (liver), 25.7 kBq/ml (lesion), and 2.1 kBq/ml (background).

### PET/CT data acquisition

All data were acquired on a Siemens Biograph mCT (Siemens Healthcare GmbH, Erlangen, Germany) in step-and-shoot mode [5]. Depending on the patient size, 6–8 bed positions were measured 1 h post injection of ^18^F-FDG (4 MBq/kg body weight) in head-first supine (HFS) position. List-mode PET data were acquired at each bed position for 120 s or 360 s (depending on the expected motion within the bed position). During PET data acquisition, a respiratory signal was recorded using the ANZAI belt system attached to the waist of the patient (ANZAI Medical Co., LTD, Tokyo, Japan). The acquired signal was used for consecutive gating. As part of the PET/CT procedure, a spiral CT scan was performed at end-expiration (100–120 kVp, pitch 1.2, table speed 92 mm/s).

**Fig. 1:**
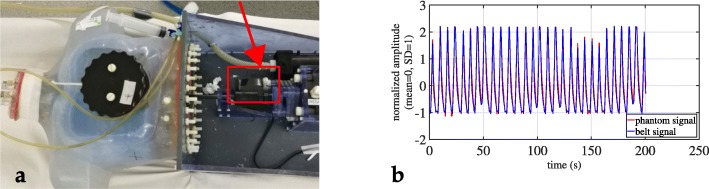
Anthropomorphic thorax phantom for cardio-respiratory motion simulation in tomographic imaging [18]. **a** Attached ANZAI pressure sensor. **b** Normalized respiratory signals

PET data of the phantom were acquired as specified in Table 1. The static acquisition at maximum expiration served as GT without respiratory motion. All other data were acquired under the influence of respiratory motion (either 1 cm and 2 cm amplitude). A respiratory signal was recorded using a ANZAI pressure sensor. The ANZAI sensor was attached to the respiratory driver as shown in Fig. 1a. Representative respiratory signals are shown in Fig. 1b. After preparation, the phantom was placed on the PET/CT bed and static and dynamic PET acquisitions were performed in HFS position. A spiral CT was acquired in end-expiration phase (parameters—120 kVp, pitch 0.9, table speed 69 mm/s).

**Table 1:** Thorax phantom settings

**Respiratory motion**	**Motion amplitude**	**Duration**
End-expiration	–	10 min
4 s exp / 3 s insp	2 cm	30 min
4 s exp / 3 s insp	1 cm	30 min

CT data with a matrix size of 512 × 512 and pixel spacing of 1.523 × 1.523 mm^2^ were used for PET attenuation correction.

### Image reconstruction algorithms

All acquired PET list-mode data were reconstructed with two different reconstruction algorithms both compensating for respiratory motion using motion estimation techniques. The first algorithm has been implemented within the e7 research framework provided by Siemens Healthcare, while the second algorithm EMrecon has been developed in-house [19]. All reconstructions make use of an ordinary Poisson ordered subsets expectation maximization algorithm (OP-OSEM) with 21 subsets and 3 iterations [20]. All data were normalized and corrected for attenuation, scatter, dead time, decay, and randoms. Furthermore, a 5-mm FWHM Gaussian filter was applied post-reconstruction. Reconstructed image sizes amounted to 200 × 200 × 109 voxels per bed position with voxel size of 4.07 × 4.07 × 2.03 mm^3^. Besides elastic motion correction, both reconstruction algorithms were also used to perform non-motion-corrected (NOMC) and OG reconstructions. For the latter, the narrowest breathing amplitude window with 35% of all data was determined from the respiratory signal, and only the list-mode data acquired within this window were reconstructed into a single 3D image [21–23].

#### Gate-to-gate motion correction

**Fig. 2:**
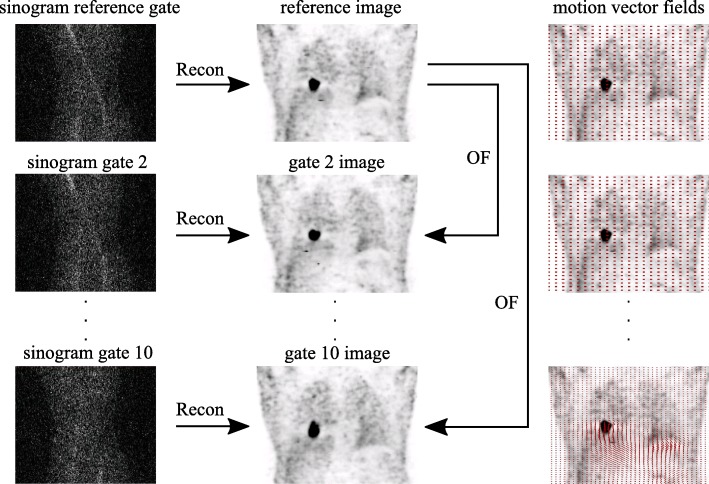
Gate-to-gate motion correction (G2G-MC)

The gate-to-gate motion correction (G2G-MC) has been implemented within the in-house-developed framework EMrecon. During this reconstruction process, 10 respiratory gates *g* were computed using an adaptive amplitude-based respiratory gating based on the ANZAI respiratory signal [23]. To this end, the amplitude range of the respiratory signal was divided into amplitude intervals (gates) with an equal amount of data each. These PET data were then reconstructed into 10 images without scatter and attenuation correction providing data for motion estimation (Fig. 2). MVFs *M*_*g*_, *g*=1,...,10, were calculated between each respiratory gate and the reference gate (end-expiration phase) with help of optical flow (OF) [24]. Using the derived MVFs, a final motion-corrected image *I* was reconstructed incorporating elastic motion correction at the level of the system matrix [15]. To this end, all LORs *l* are elastically deformed according to the motion information of the MVFs *M*_*g*_ and are then further processed during image reconstruction, leading to the following iterative EM reconstruction scheme:

1 $$ I^{n+1 }(b)=I^{n }(b)\frac{ 1 }{ \sum_{g }{ M_{g}^{T}}\left(B \left(\frac{ 1 }{ A(l)N(l)} \right) \right)} \sum_{g }{ M_{g}^{T}}\left(B \left(\frac{ P(l,g) }{ F(M_{g}(I^{n} (b)) + O(l,g)} \right) \right),   $$

with *n* denoting the iteration number, *b* the image voxels, *P* the number of prompt events, *A* the attenuation correction factors (ACF), *N* the normalization factors, *O* denoting (randoms*norm+scatter)*ACF, *F* and *B* the forward and backward projectors, respectively, and $M_{g}^{T}$ the transpose of *M*_*g*_.

#### Elastic motion correction with motion deblurring

**Fig. 3:**
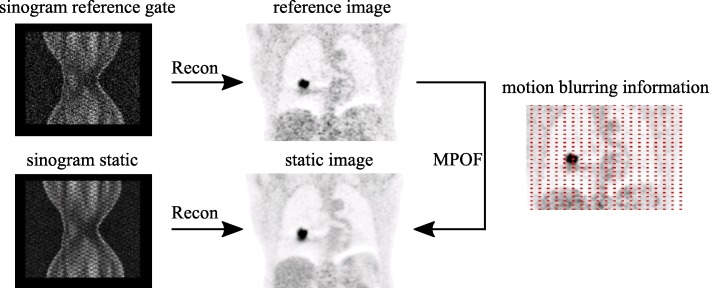
Motion correction using motion deblurring (DEB-MC) [26]

DEB-MC is implemented in the e7 toolbox as the research version of the clinically available product “OncoFreeze” from Siemens Healthcare (Knoxville, TN, USA) [25, 26]. Briefly, this method uses a single deblurring kernel *M* during iterative image reconstruction, where the motion blurring between the reference image (OG) and the static reconstruction is estimated using mass-preserving optical flow (MPOF) (Fig. 3) [27, 28]. This leads to the following EM reconstruction scheme:

2 $$ I^{n+1 }(b)=I^{n }(b)\frac{ 1 }{ M^{T}\left(B \left(\frac{ 1 }{ A(l)N(l)} \right) \right)} { M^{T}}\left(B \left(\frac{ P(l) }{ F(M(I^{n} (b)) + O(l)} \right) \right).   $$

### Image analysis

To evaluate the effect of MC on quantitative data, volume-of-interest (VOI) defined on multiple lesions were analyzed, and changes (ratios of MC/NOMC) in standardized uptake values (SUV _max_, SUV _mean_) and the metabolic ^18^F-FDG volume (MV) were reported. For tumour segmentation, SUV _mean_ and the metabolic volume are based on a 50% threshold of SUV _max_ of each lesion:

3 $$ \mathrm{SUV_{threshold}} = 0.5~\times~\mathrm{{SUV}_{max}(lesion)}.   $$

This threshold was applied voxel-wise to volumes that completely cover the tumour of interest resulting in SUV _mean_ and MV.

For the phantom study, mean (MEAN) and maximum (MAX) activity concentrations were calculated together with the recovery coefficients (RC) between the reconstructed images and GT (at maximum expiration). To this end, MVs were defined on the GT images for both, e7 and EMrecon. These MVs were also used to analyze the corresponding NOMC and MC data. The RC was defined as the mean activity concentration divided by the mean activity concentration of the corresponding GT data.

As image quality parameter, the contrast-to-noise ratio (CNR) of the lesions was analyzed using the following definition (SD = standard deviation). The background region was placed directly beside the respective lesion.

4 $$ \text{CNR} = \frac{\mathrm{{SUV}_{mean}(lesion)-{SUV}_{mean}(background)}}{\mathrm{SD(SUV(background))}}.   $$

### Statistical analysis

For the statistical evaluation of differences in outcome between G2G-MC and DEB-MC, MC/NOMC ratios of SUV _max_, SUV _mean_, MV, and CNR were analyzed with Wilcoxon signed-rank tests using Matlab (version 9.3 R2017b, The Mathworks Inc., Natick, MA, USA). *p* values <0.05 were considered as statistically significant.

## Results

### Phantom data evaluation

**Fig. 4:**
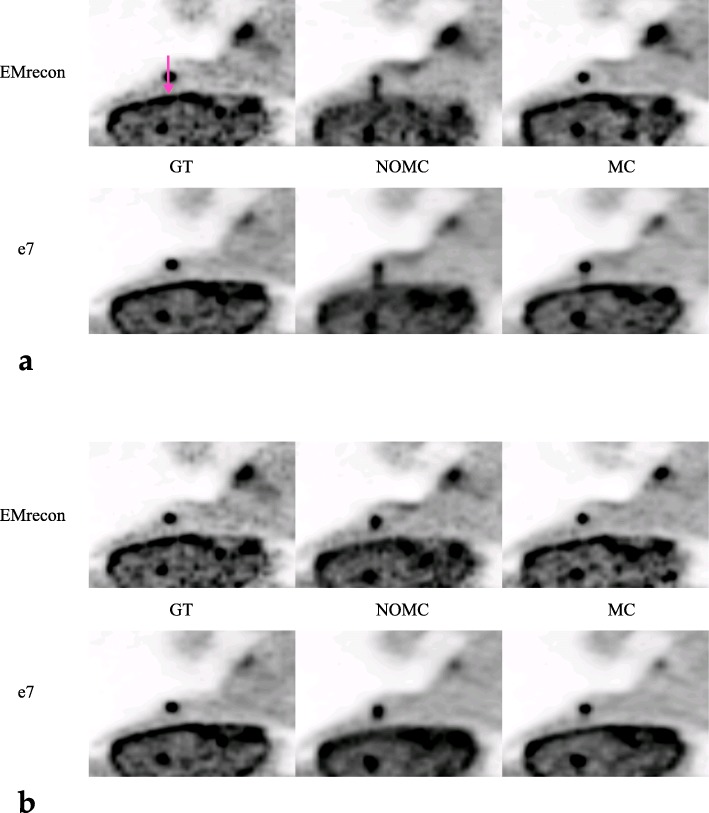
Thorax phantom experiments with different motion amplitudes (**a** 2 cm, **b** 1 cm) showing GT results in comparison to NOMC and MC (coronal planes)

By visual inspection of the different motion and non-motion-corrected images (Fig. 4), both motion correction methods show a clear effect on the MC images with sharper delineation of the lesion compared to the NOMC images. As expected, a larger lesion displacement of 2 cm leads to more visible blurring compared to the mild motion extent of 1 cm.

**Fig. 5:**
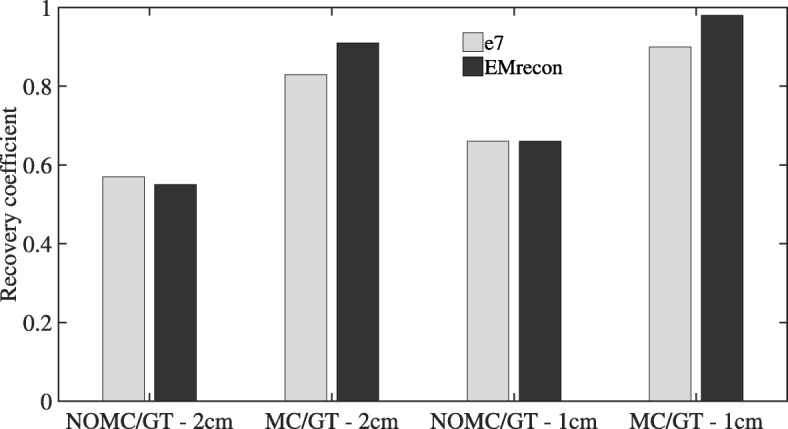
Recovery coefficients of the lesion evaluation for the phantom data

**Fig. 6:**
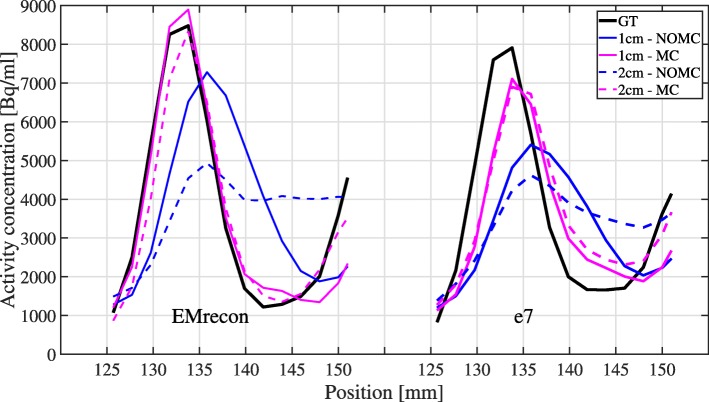
Line profiles (sagittal plane, see arrow in Fig. 4) through the lesion for EMrecon (left) and e7 (right)

MVs of the GT acquisitions result in 0.807 ml (e7) and 0.740 ml (EMrecon), respectively. Both motion-corrected reconstructions using DEB-MC and G2G-MC show similar improvements in quantitative lesion activity (Table 2) for both motion amplitudes. The effect of motion correction can also be shown quantitatively in the RCs (Fig. 5). Both MC methods show high recovery values of 91% (2 cm) and 98% (1 cm) for G2G-MC and 83% (2 cm) and 90% (1 cm) for DEB-MC. G2G-MC shows slightly higher RCs in this single case examination, and this is also visible in the profile analysis (Fig. 6).

**Table 2:** Activity concentrations of phantom data for all reconstructions

			**2 cm motion**		**1 cm motion**	
	Parameter	GT	NOMC	MC	NOMC	MC
e7	Max (Bq/ml)	8474	4621	7599	6221	9028
	Mean (Bq/ml)	5990	3409	4957	3969	5412
EMrecon	Max (Bq/ml)	9452	5054	9342	7280	9310
	Mean (Bq/ml)	6527	3579	5915	4324	6397

### Patient data evaluation

**Fig. 7:**
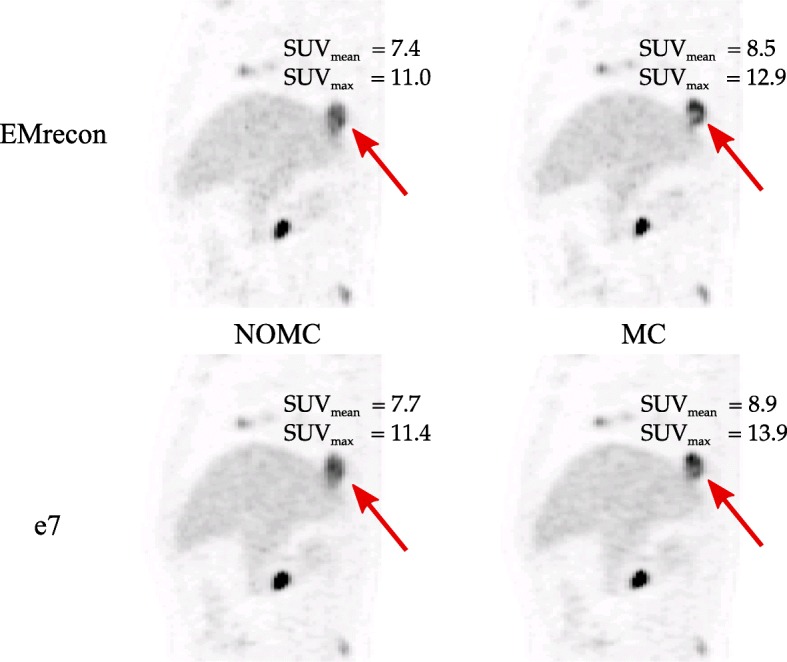
Patient case *#*1 (sagittal plane) showing NOMC and MC reconstructions. The arrow highlights motion correction effects

**Fig. 8:**
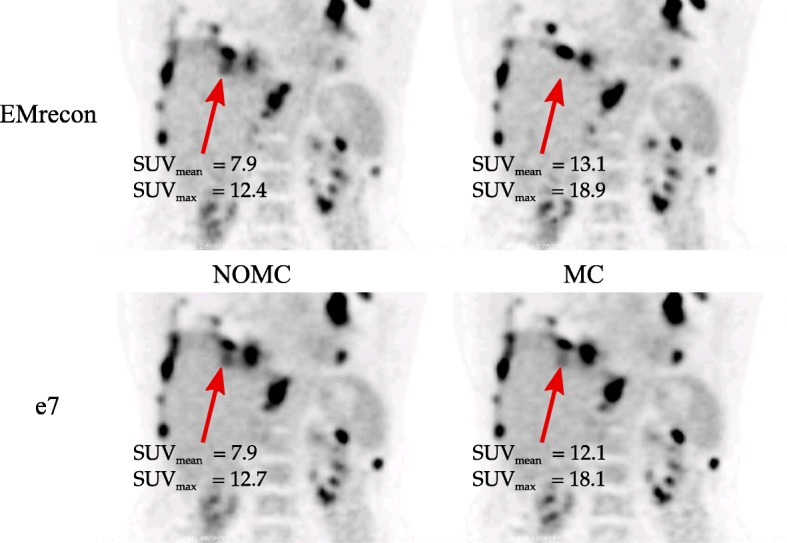
Patient case *#*5 (coronal plane) showing NOMC and MC reconstructions. The arrow highlights motion correction effects

Figures 7 and 8 exemplarily show NOMC and MC reconstructions of representative patient data sets. The first case (Fig. 7) demonstrates visible improvements (red arrows) after performing MC for both e7 and EMrecon algorithms. In a second case (Fig. 8), similar enhancements could be found for both algorithms, while DEB-MC seemed to under-correct a singular lesion with large motion amplitude (red arrow).

#### Descriptive analysis

Similar to the phantom evaluation, the patient study showed clear increases in SUV _max_ and SUV _mean_ in correspondence with a decrease in MVs after MC (Table 3). MC led to an average increase of 11% in SUV _max_ and 12% in SUV _mean_ after G2G-MC, and of 12% after DEB-MC, respectively. The average decrease in MVs was 18% for G2G-MC, and 28% for DEB-MC. Both MC reconstructions provided higher CNRs than the NOMC reconstructions (24% higher after G2G-MC, 8% after DEB-MC, Table 3).

**Table 3:** Mean ± SD for SUV _max_, SUV _mean_, MV, and CNR of all lesions reconstructed with and without MC

	**e7**		**EMrecon**	
Parameter	NOMC	DEB-MC	NOMC	G2G-MC
**SUV**_**max**_	8.41 ± 6.33	9.57 ± 7.47	8.71 ± 6.52	9.77 ± 7.38
*p* vs NOMC	–	< 0.0001	–	< 0.0001
**SUV**_**mean**_	5.65 ± 4.49	6.40 ± 5.19	5.82 ± 4.54	6.56 ± 5.13
*p* vs NOMC	–	< 0.0001	–	< 0.0001
**MV (ml)**	6.42 ± 8.67	4.59 ± 5.98	5.72 ± 7.77	4.41 ± 5.79
*p* vs NOMC	–	< 0.0001	–	< 0.0001
**CNR**	27.8 ± 37.4	31.6 ± 41.1	24.5 ± 30.4	28.1 ± 31.5
*p* vs NOMC	–	< 0.0001	–	< 0.0001

#### Statistical analysis

**Fig. 9:**
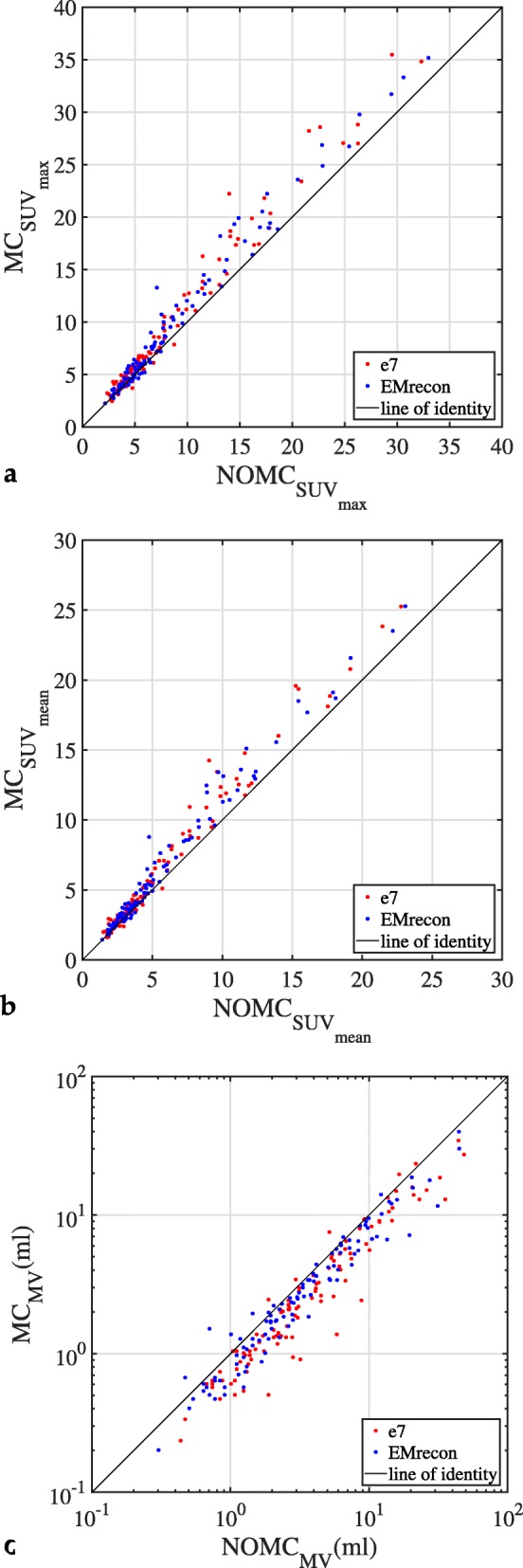
Scatter plots for **a** SUV _max_, **b** SUV _mean_, and **c** MV comparing e7 and EMrecon reconstructions before and after MC

The statistical analysis found significant differences (*p* < 0.0001) between NOMC and MC reconstructions for SUV _max_, SUV _mean_, MV, and CNR for both, EMrecon and e7 (Table 3). These statistical findings are also detectable in the scatter plot comparison of all individual results (Fig. 9). Furthermore, no significant differences were found comparing the ratios of MC and NOMC reconstructions for SUV _max_, SUV _mean_, and CNR, but for the MV (Table 4).

**Table 4:** Mean ± SD of MC/NOMC ratios for SUV _max_, SUV _mean_, MV, and CNR of all lesions

**Parameter**	**e7**	**EMrecon**
**SUV**_**max**_**(MC)/SUV**_**max**_**(NOMC)**	1.12 ± 0.14	1.11 ± 0.14
*p* vs EMrecon	0.343	–
**SUV**_**mean**_**(MC)/SUV**_**mean**_**(NOMC)**	1.12 ± 0.14	1.12 ± 0.14
*p* vs EMrecon	0.615	–
**MV(MC)/MV(NOMC) (ml)**	0.73 ± 0.21	0.82 ± 0.22
*p* vs EMrecon	0.00023	–
**CNR(MC)/CNR(NOMC)**	1.08 ± 0.62	1.24 ± 0.94
*p* vs EMrecon	0.747	–

## Discussion

In this study, for the first time, a direct comparison of the fast elastic motion correction technique DEB-MC with state-of-the-art elastic motion correction G2G-MC was performed within a clinical patient population and advanced phantom scans. Both MC techniques for PET/CT data were applied to 28 clinical cases with a total of 107 identified lesions.

The direct comparison of DEB-MC and G2G-MC demonstrated similar performance resulting in clear image enhancements and quantitative improvements (Figs. 7 and 8). Both methods led to a highly significant increase in SUVs and CNR and a decrease in MVs. For both MC approaches, the lesion contrast represented by CNR showed clear contrast enhancements after motion correction.

DEB-MC seems to be a suitable alternative for G2G-MC in whole-body PET motion correction. Additionally, DEB-MC is highly advantageous with regard to the overall reconstruction time. While G2G-MC estimates the MVFs between each gate and a reference gate and therefore has to pre-reconstruct all respiratory gated images, DEB-MC only needs to estimate the deblurring kernel between the OG and the NOMC reconstructions, resulting in significant time savings with DEB-MC being 10 times faster than G2G-MC. In addition, G2G-MC needs to calculate MVFs for all gates, whereas DEB-MC just performs a single MVF calculation which could be more stable due to enhanced statistics. Thus, DEB-MC seems to be easier applicable in the clinical workflow leading to only mildly increased reconstruction times compared to standard NOMC reconstructions.

In a few clinical situations, DEB-MC did not fully correct all motion effects as can be visualized and quantified on phantom (Fig. 4) and patient data (Fig. 8). There are two explanations for this finding. First, the motion amplitude may be too large to be represented by a single blurring kernel between the OG and the rest of the data. Both cases show very large motion displacements with amplitudes in the range of 2 cm which supports this hypothesis. Second, non-physiological or very irregular motion patterns may lead to OG definitions at a respiratory phase which may be outside end-expiration. Also here, a single blurring kernel may not describe all motion effects leading to images with some residual motion. Although these effects may degrade image quality in a few clinical cases, it is assumed that still most of the motion is correctly assigned which justifies the use of the method. This finding should be further investigated. It has been reported before that motion correction may lead to degraded image results for small motion amplitudes [29]. Although this effect was not expected in both motion correction methods used in our study, we tested G2G-MC with a zero MVF which resulted in no measurable image degradation.

This study is limited by the fact that all EMrecon reconstructions show higher noise levels compared to e7-based reconstructions. We assign this finding to two possible reasons: firstly, the EMrecon reconstruction is using uncompressed sinograms of size 400 × 168 × 621 for image reconstruction while e7 reconstructions were performed on compressed sinograms of size 200 × 168 × 621. This compression will reduce image noise and produce smoother images compared to the uncompressed situation. Secondly, both methods use different ray tracing projector techniques which may have an impact on the resulting noise level. Since a direct quantitative comparison of the two reconstruction techniques lies outside the scope of this study, we focused our study on the evaluation and comparison of motion correction effects rather than the true activity levels. As the noise level on EMrecon reconstructions is higher than on e7 reconstructions, DEB-MC showed in general slightly larger MVs than G2G-MC. This is due to the fact that the MVs are defined with a threshold based on the local SUV _max_ of the tumour region. However, this statistical difference should not affect the MC/NOMC ratios showing the enhancement in tumour uptake due to motion correction. Here, both motion correction methods show comparable improvements, which implies an equivalent application of both methods in clinical PET/CT.

## Conclusion

Both evaluated motion correction techniques, fast elastic motion compensation based on motion de-blurring (DEB-MC) and elastic motion correction based on gate-to-gate motion estimation (G2G-MC), are equally applicable to clinical whole-body PET/CT data leading to quantitative improvements in SUV _max_, SUV _mean_, and MVs. SUV analysis showed no significant differences for the MC/NOMC ratios for both approaches but significantly higher SUVs after MC reconstructions. Both MC methods clearly improve lesion contrast and are therefore a valuable tool for improved whole-body PET imaging.

## Data Availability

All data and materials used within this study are available on reasonable request.

## Abbreviations

^18^F: fluorine-18
ACF: Attenuation correction factors
CNR: Contrast-to-noise ratio
CT: Computed tomography
DDG: Data-driven gating
EM: Expectation maximization
DEB-MC: Deblurring motion correction
FDG: Fluorodeoxyglucose
FWHM: Full width at half maximum
GT: Ground truth
HFS: Head first: supine
LOR: Lines-of-response
MC: Motion-corrected, motion correction
G2G-MC: Gata-to-gate motion correction
MPOF: Mass-preserving optical flow
MV: Metabolic volume
MVF: Motion vector field
NOMC: Non-motion-corrected, no motion correction
OF: Optical flow
OG: Optimal gate
OP-OSEM: Ordinary Poisson ordered subset expectation maximization
PBG: Phased bin gating
PET: Positron emission tomography
RC: Recovery coefficient
SD: Standard deviation
SNR: Signal-to-noise ratio
SUV _max_: Standardized uptake value (maximum)
SUV _mean_: Standardized uptake value (mean)
TOF: Time-of-flight
VOI: Volume-of-interest
